# A Genetic Screen To Identify Genes Influencing the Secondary Redox Couple NADPH/NADP^+^ in the Yeast *Saccharomyces cerevisiae*

**DOI:** 10.1534/g3.119.400606

**Published:** 2019-11-22

**Authors:** Shambhu Yadav, Tejasvinee Atul Mody, Archi Sharma, Anand Kumar Bachhawat

**Affiliations:** Department of Biological Sciences, Indian Institute of Science Education and Research, Mohali, S.A.S. Nagar, Punjab 140306, India

**Keywords:** NADPH, Glutathione, *Saccharomyces cerevisiae*, Mitochondria, Genetic screen

## Abstract

NADPH is an important cofactor in the cell. In addition to its role in the biosynthesis of critical metabolites, it plays crucial roles in the regeneration of the reduced forms of glutathione, thioredoxins and peroxiredoxins. The enzymes and pathways that regulate NADPH are thus extremely important to understand, and yet are only partially understood. We have been interested in understanding how NADPH fluxes are altered in the cell. We describe here both an assay and a genetic screen that allows one to discern changes in NADPH levels. The screen exploits the secondary redox property of NADPH. At low levels of glutathione we show that the redox contributions of NADPH become critical for growth, and we have used this to develop a genetic screen for genes affecting NADPH homeostasis. The screen was validated in pathways that both directly (pentose phosphate pathway) and indirectly (glycolytic pathway) affect NADPH levels, and was then exploited to identify mitochondrial genes that affect NADPH homeostasis. A total of 239 mitochondrial gene knockouts were assayed using this screen. Among these, several genes were predicted to play a role in NADPH homeostasis. This included several new genes of unknown function, and others of poorly defined function. We examined two of these genes, FMP40 which encodes a protein required during oxidative stress and GOR1, glyoxylate reductase. Our studies throw new light on these proteins that appear to be major consumers of NADPH in the cell. The genetic screen is thus predicted to be an exceedingly useful tool for investigating NADPH homeostasis.

NADPH (along with its oxidized form NADP^+^) is an important cofactor in the cell. NADPH/NADP^+^ together with NADH/NAD^+^ participates in about 2000 biochemical reactions in the cell, thought to represent approximately about 25% of all biochemical reactions (Opitz and Heiland 2015).

NADPH, as a cofactor, plays a crucial role in regenerating reduced glutathione, oxidized thioredoxins and oxidized peroxiredoxins. Furthermore, it participates in many biosynthetic reactions requiring reducing equivalents such as in the synthesis of fatty acids, sterols, and DNA (Ying 2008). NADPH, surprisingly, also has an oxidant role since it is a substrate of NADPH oxidase generating superoxides either for signaling purposes or in an anti-inflammatory response. In mammalian cells, NADPH also plays a non-redox role as a precursor for NAADP (Nicotinic acid adenine dinucleotide phosphate) which is very critical for calcium mobilization and in the regulation of calcium homeostasis (Ali *et al.* 2016; Yang and Sauve 2016).

Recent tracer experiments have been very valuable in deciphering the contributions and compartmentalization of the main pathways of NADPH generation (Lewis *et al.* 2014). However, additional pathways are possibly contributing to the NADPH pools. Currently, there are limited studies that have examined the additional NADPH generation pathways or derived strategies to identify the genes involved. Therefore further approaches are required to identify the different potential genes that affect the levels of these metabolites, to get a more complete picture. Given the importance of NADPH in cellular bioenergetics, signaling and redox, knowledge of the gamut of the reactions and their relative importance under different conditions can be utilized to increase the general NADPH pools. Although a genetic strategy in yeast was attempted to identify genes suppressing defects in the key NADPH synthesizing enzyme, Zwf1p, it led to limited success owing to the stringent nature of the screen (Grabowska and Chelstowska 2003).

Owing to the high concentrations (millimolar levels) of glutathione, the contributions of NADPH in redox homeostasis remains masked and the factors determining their pools become difficult to identify. In this study, we have sought to devise strategies to understand the genes and pathways that could influence the pools of NADPH by deliberately lowering the intracellular glutathione concentrations.

Although glutathione is essential for Fe-S loading (requiring only micromolar glutathione concentrations), the bulk function of glutathione is to perform redox functions and detoxifications (which require high glutathione concentrations). The essential and bulk functions of glutathione are separable (Kumar *et al.* 2011). Using glutathione biosynthetic mutants, it seems possible, therefore that if glutathione is provided in micromolar concentrations, we could meet the essential requirement of glutathione without having high levels that would mask the contributions of the secondary redox couples. In this low glutathione background, genes and mutations affecting NADPH pools could then be identified and investigated.

In this manuscript, we describe our efforts to investigate and validate this hypothesis, and following the validation we have subsequently used this to set up a genetic screen to identify and investigate new players affecting NADPH levels. Our studies indicated that it is indeed possible to set up a sensitive assay for detecting NADPH levels in the cell. The genetic screen enabled us to screen the mitochondrial knockout collection to determine major contributors and consumers of NADPH. Several of the genes were of unknown or poorly characterized function. Two of these, that were among the major consumers of NADPH in the mitochondria, Fmp40p and Gor1p were investigated in detail to obtain insights into their role in NADPH homeostasis.

## Materials and Methods

### Chemicals and reagents

Chemicals used in this manuscript were of analytical grade and purchased from commercial sources. Growth medium components were purchased from Difco, Sigma–Aldrich, and HiMedia. All primers were purchased from Integrated DNA Technologies (IDT) India and Sigma India. Vent DNA polymerase and restriction enzymes were purchased from NEB (New England Biolabs). Plasmid miniprep and DNA gel-extraction kits were obtained from Bioneer and Promega. PVDF blotting membrane purchased from GE Healthcare Life science, USA. Western HRP substrate was obtained from Millipore, India. NADPH estimation kit was obtained from Promega.

### Growth of Saccharomyces cerevisiae

*Saccharomyces cerevisiae* was routinely maintained on YPD media. For growth assays, *S. cerevisiae* strains or transformants carrying the plasmid were grown for 8-12 hr in SD medium excluding selection markers and reinoculated in fresh selection medium to an OD_600_ = 0.1 and grown for 7-8 hr. The exponential growth phase cells were harvested, washed twice and resuspended in double distilled sterile water to an OD_600_ = 0.2. These were serially diluted into 1:10, 1:100, and 1:1000. 10 μl of the cell resuspensions were spotted on SD medium containing different concentrations of GSH, and Met depending upon selection markers, as a sole sulfur source. The plates were incubated at 30° and images captured after 3-4 days.

### Total NADPH measurement by luminescence-based kit (Promega)

For total NADPH estimation, *S. cerevisiae* WT or mutant cells were grown in SD medium containing 200 µM GSH at 30° for 12 hr and reinoculated in fresh SD medium without GSH at initial OD_600_ = 0.2; cells were allowed to grow at 30° till the early exponential growth phase OD_600_ = 0.6 – 0.8, with shaking at 220 rpm. Equal number of cells (OD_600_ = 1) were harvested at 5000 rpm and washed with sterile water followed by resuspension of the cells in lysis buffer (100 mM KH_2_PO_4_, 1.2 M Sorbitol). Spheroplasts were prepared by adding the Zymolase at the final concentration of 0.3 mg/mL and subsequently incubating at 30° in shaking incubator at (100 rpm) for 1 hr. The spheroplasts were resuspended in 100 µL lysis buffer and an equal volume of the reaction mixture was added from NADP/NADPH-Glo assay kit. The reaction mixture was incubated at room temperature for 45 min, and readings were taken using luminescence spectrometer. Data were analyzed using GraphPad Prism 5.0.

### Gene disruption in yeast Saccharomyces cerevisiae

The GSH1 gene was disrupted in mitochondrial and mitochondrial-associated genes of *S. cerevisiae* strains using *gsh1*::*LEU2* disruption cassette containing plasmid (ABE523). The plasmid was digested with HindIII restriction enzyme and transformed in all mitochondrial or other pathways genes individually. The *gsh1* gene deletion was selected by leucine prototrophy and confirmed by glutathione auxotrophy. The FMP40 gene was disrupted in different *S. cerevisiae* gene deletion strains using the *fmp40*::*HIS3* disruption cassette containing plasmid. The disruption cassette was excised from the plasmid by digestion with BamHI and StuI restriction enzymes and transformed into different *S. cerevisiae* deletion strains. The *fmp40* disruptions were selected by histidine prototrophy.

### Recombinant protein expression and purification of 6XHis-tagged Gor1 protein

For Gor1 protein purification, a c-terminal 6XHis-tagged GOR1 gene was transformed in BL21 (DE3) *E. coli* strain. The primary culture was grown overnight in LB media with ampicillin 100 μg/ml for selection. The secondary culture was inoculated at OD_600_= 0.05 and allowed to grow until OD_600_ reached 0.6. Induction was given at OD_600_= 0.6 with 1mM IPTG and incubate at 30° for 5 hr, 220 rpm. Cells were harvested at 8000 rpm for 5 min at 4° and the supernatant was discarded followed by washing with distilled water at 8000 rpm. The cells were lysed by sonication using lysis buffer with 15 sec ON and 30 sec OFF cycle and 20% amplitude and 15 cycles. The sonicated lysate was centrifuged at 10000 rpm for 20 min at 4°. The supernatant was incubated with washed Ni-NTA beads for 2 hr at 4°. The protein-bound beads were subjected to three washes with washing buffer followed by centrifugation at 1500 rpm for 5 min at 4°. The Gor1p protein was eluted with 300 mM Imidazole. The 10 kD concentrator (Millipore) was used to remove Imidazole by buffer exchange with phosphate buffer (50mM Sodium phosphate monobasic, 300 mM NaCl and 10% glycerol). After buffer exchange, the purity of the protein was checked on 10% SDS-PAGE and Gor1 protein concentration was checked by NanoDrop and Bradford assay, and the protein was flash-frozen in liquid nitrogen and stored at -80° for further use.

### Gor1p activity and kinetic studies

The activity of the Gor1p against various substrates (glyoxylate, hydroxypyruvate, glycolate, pyruvate, alanine, and glycine) was evaluated. The reaction was carried out in a 1 mL quartz cuvette containing 50 mM Potassium phosphate (pH 7.4) with different concentration of substrate (0-40 mM). The reaction was initiated by adding 0.25 mM NADPH. The reactions were measured in triplicates with two biological replicates. The reaction was monitored at 30° by following NADPH absorbance at 340 nm using Perkin Elmer UV/Vis Lambda 25 Spectrophotometer. The results were represented as enzymatic activities *vs.* substrate concentration (mM) and all analysis was done using GraphPad Prism 5.

### Statistical analysis

Statistical analyses were performed using GraphPad Prism5. The average value and standard error of the mean (s.e.m.) were calculated.

### Data availability

Strains and plasmids are available upon request. Supplemental material available at figshare: https://doi.org/10.25387/g3.9976517.

## Results

### Yeast strains with suboptimal glutathione levels are sensitive to changes in NADPH levels as seen through overexpression and knockouts of NADPH generating enzymes

Glutathione, which is found in millimolar concentrations in the cells is essential for the growth of *S. cerevisiae*. The high concentrations of glutathione, which are required for its redox functions are likely to mask the contributions of the secondary redox couples which are found at much lower concentrations in the cell. As we were interested in identifying genes that influence the NADPH levels in the cell, we sought to identify conditions in the cell wherein the contributions of NADPH levels would become significant. Since NADPH is involved in the recycling of oxidized glutathione and thioredoxins that are critical for redox homeostasis, it is possible that in cells with only limiting levels of glutathione, small changes in NADPH levels might be detectable.

**Figure 1:**
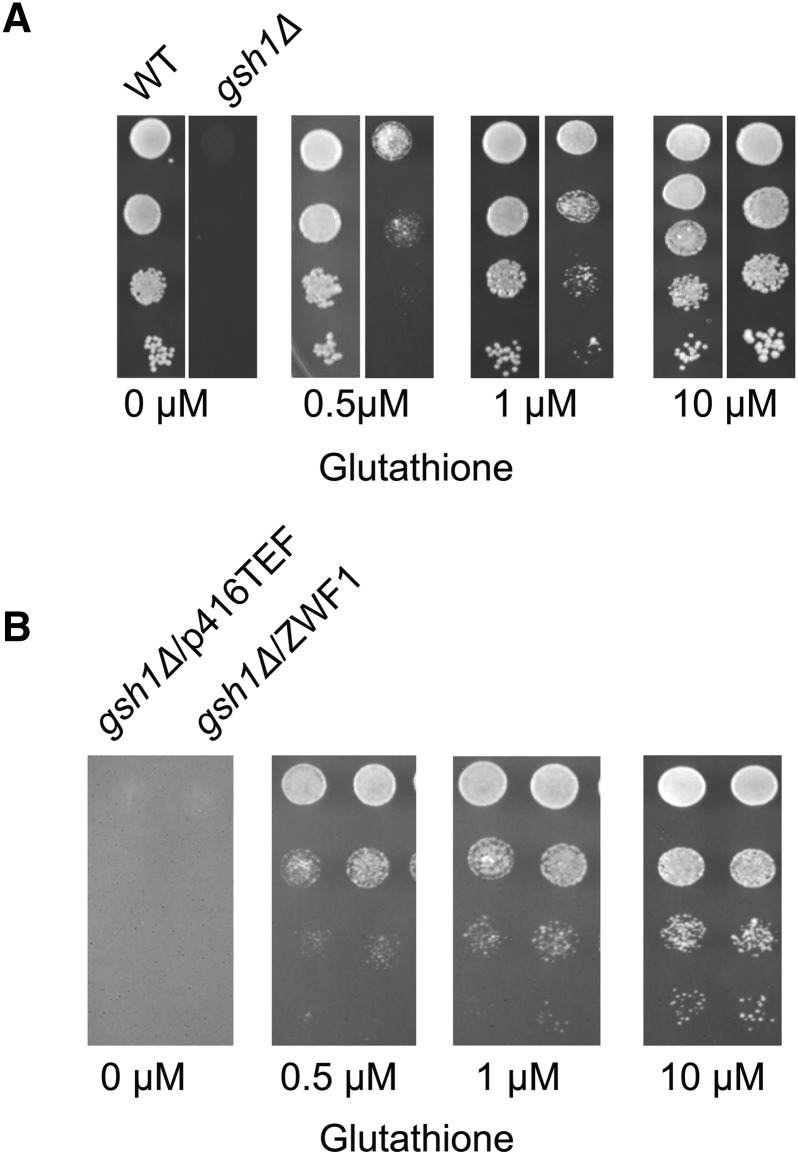
(A) Identification of glutathione concentrations for sub-optimal growth of S. cerevisiae *gsh1Δ*. S. cerevisiae *gsh1Δ* was initially grown in minimal medium containing 200 μM reduced glutathione (GSH), washed twice with sterile water, and reinoculated in minimal medium without GSH for 7-8hrs. Cells were harvested, washed, resuspended in sterile water and serially diluted to give 0.2, 0.02, 0.002, and 0.0002 OD_600_ of cells. 10 μL of the cells were spotted on minimal medium containing a different concentration of GSH. (B) Growth comparison of *gsh1Δ* overexpressing ZWF1. Growth and serial dilution of *gsh1Δ* bearing the TEF-ZWF1 transformants described above Figure 1(A).

As a first step toward this goal, we sought to determine the amount of external glutathione required for sub-optimal growth of a mutant deficient in glutathione biosynthesis, *(gsh1∆)*. We observed that at glutathione concentrations as low as 10 µM, the *gsh1∆* cells grew very well and higher concentrations did not improve the growth. At lower concentrations of 0.5-1 µM, we observed that growth of *gsh1∆* cells was partial, suggesting that glutathione was becoming limiting (Figure 1A). These limiting glutathione concentrations thus appear to be most suitable to unmask and assess changes in concentrations of other redox couples such as NADPH/NADP^+^. To assess this possibility, we examined the effects of deleting or overexpressing genes known to generate NADPH.

The oxidative part of the pentose phosphate (PP) pathway is the main source of the reduced form of NADPH. The first step of the oxidative PP pathway is catalyzed by glucose 6-phosphate dehydrogenase (Zwf1p), which is known to be a key enzyme for NADPH generation. We created a GSH1 deletion in a *zwf1∆* mutant background (*gsh1∆zwf1∆*) and compared this to the growth of a single *gsh1∆* strain at low concentrations of glutathione. Although we observed that *gsh1∆zwf1∆* shows a greater growth defect than the single *gsh1∆* background on low glutathione, as the *zwf1Δ* itself has a significant growth defect, it became difficult to draw conclusions from the deletions and comparisons with the *gsh1Δ* where ZWF1 is not deleted. We therefore focused on ZWF1 overexpression experiments.

ZWF1 overexpression is well known to increase cellular NADPH levels. Therefore we wanted to determine if overexpression of ZWF1 could enhance the growth of the *gsh1∆* strain at low glutathione conditions. We observed that ZWF1 expression downstream of the strong TEF promoter did indeed lead to a significantly enhanced the growth of *gsh1∆* cells under these conditions (Figure 1B).

These experiments indicate that our assay could be sufficiently sensitive for unmasking NADPH homeostatic genes.

### Deletion of NADPH consuming alcohol dehydrogenase enzyme leads to improved growth in the genetic screen

**Figure 2:**
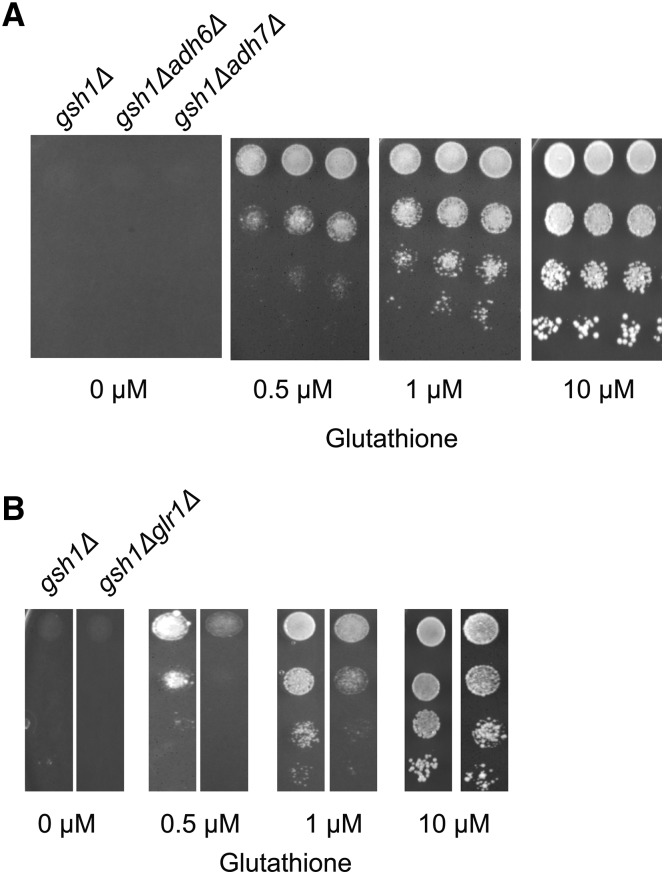
Growth of deletions in NADPH consuming enzymes carrying deletions in *gsh1Δ* (A). Comparison of deletions of Alcohol dehydrogenases, ADH6 and ADH7 in *gsh1Δ* background at different glutathione concentrations. Growth and serial dilution of *adh6Δgsh1Δ* and *adh7Δgsh1Δ* performed as described in legends to Figure (1A) (B) Comparison of deletions of Glutathione reductase, GLR1, in *gsh1Δ* background. Growth and serial dilution of *glr1Δgsh1Δ* performed as described in legends to Figure (1A).

To further examine the validity of the genetic screen, we evaluated the growth of knockouts of genes coding for enzymes that are known to utilize NADPH as a cofactor. ADH6 and ADH7 are NADPH-dependent medium chain alcohol dehydrogenases. We performed growth based phenotypic assays with strains carrying a deletion in either of these two enzymes in the *gsh1Δ* background. We observed that both these double deletions (*adh6Δgsh1Δ* and *adh7Δgsh1Δ*) showed improved growth relative to *gsh1Δ* at low glutathione concentration (Figure 2A). Therefore, our genetic screen is successfully able to detect consumers of NADPH.

Glutathione reductase (GLR1) catalyzes the conversion of oxidized glutathione to its reduced form, is also a key consumer of NADPH. Under low glutathione condition, Glr1p is expected to be very critical to maintain the shift of the equilibrium from oxidized to reduced glutathione. In contrast to other consumers of NADPH, therefore, GLR1 deletion would be expected to be deleterious for growth under low glutathione conditions. To validate this aspect, we created a *glr1Δ* in the *gsh1Δ* background and performed the growth based phenotypic assays under low glutathione conditions. As expected, we observed a greater growth defect in the *glr1Δgsh1Δ* double deletion strains in comparison to the *gsh1Δ* background at low glutathione concentration (Figure 2B). These studies further underline the validity of the screen, while also indicating that the mechanistic basis for the screen might be to provide NADPH for the GLR1 enzyme that will enable the equilibrium of the minimal cellular glutathione to be pushed toward reduced glutathione.

### Indirect enhancement of the flux in the pentose phosphate pathway by knockdown of glycolytic enzymes can be detected in the genetic screen under low glutathione conditions

To determine if the genetic screen for NADPH homeostatic genes could also detect changes in NADPH levels mediated by pathways not directly involved in the generation of NADPH, we examined genes and pathways known to indirectly impact the pentose phosphate flux for NADPH generation.

Inhibition of the glycolytic pathway, through inactivation of the pathway enzymes has been shown to enhance flux in the pentose phosphate pathway (Grüning *et al.* 2014; Stincone *et al.* 2015). For instance, down regulation of pyruvate kinase (PYK1/2) has been shown to lead to accumulation of the substrate, phosphoenol pyruvate which inhibits Triose phosphate isomerase (TPI) leading to accumulation of metabolites in the upper glycolytic pathway and consequent increase in flux in the PP pathway (Grüning *et al.* 2014). The down-regulation of PYK1 and PYK2 in fact appears to trigger a switch toward oxidative phosphorylation, but the consequent increase in ROS seems to be offset by the increased flux in the PP pathway and higher NADPH levels (Grüning *et al.* 2011).

The change of the metabolic flux from glycolysis to the oxidative pentose phosphate pathway for the generation of NADPH appears to be a conserved response to oxidative stress (Ralser *et al.* 2007). The glycolytic enzyme, glyceraldehydes-3-phosphate dehydrogenase (TDH1/TDH2/TDH3) is known to be inactivated under oxidative stress (Grant 2008). Inactivating enolase1 (ENO1) also pushes the carbon flux toward the pentose phosphate pathway. This shift of the glycolytic pathway toward the PP pathway is required for the regeneration of NADPH, and appears to be a key factor in the initial cellular response to maintain redox balance (Kuehne *et al.* 2015).

**Figure 3:**
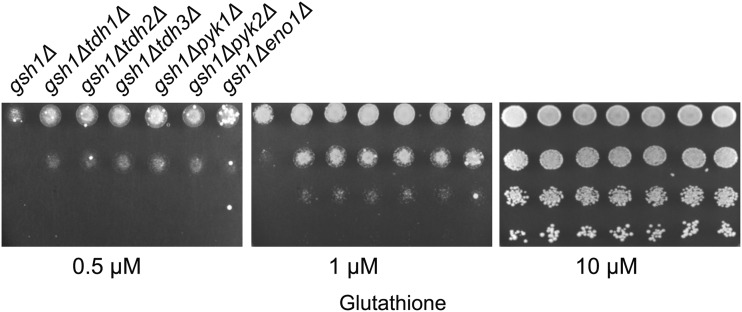
Growth of deletions of selected non-essential glycolytic genes in *gsh1Δ* background at different glutathione concentrations. Growth and serial dilution of *tdh1Δgsh1Δ*, *tdh2Δgsh1Δ*, *tdh3Δgsh1Δ*, *eno1Δgsh1Δ*, *pyk1Δgsh1Δ*, and *pyk2Δgsh1Δ* performed as described in legends to Figure (1A).

We were interested, therefore, to examine whether the genetic screen that we had developed might be able to detect changes in NADPH levels that could result from such indirect effects. To evaluate this aspect, we examined knockouts of a few key enzymes of the glycolytic pathway (in the *gsh1∆* deletion background), and investigated whether they showed improved growth (as compared to *gsh1∆)* as might be expected if these mutants were pushing greater flux into the PP pathway leading to higher NADPH generation. We created *gsh1∆* deletions in yeast strains with knockouts of glycolytic enzyme genes TDH1, TDH2, TDH3, PYK1, PYK2 and ENO1 genes and compared their growth with the single *gsh1∆* deletion under low glutathione conditions. We found that deletion of these genes in the *gsh1∆* background improved the growth of the strains relative to *gsh1∆* (Figure 3). This result suggests that our screen was indeed able to monitor the increase in flux in the PP pathway leading to the enhanced growth. The results indicate that the genetic screen was sufficiently sensitive to detect genes and enzymes that have an indirect effect on the NADPH levels.

### Knockdown of TCA enzymes and their impact on growth of strains with low glutathione content

In yeast, the TCA cycle is known to generate NADH, but NADPH generation does not appear to be a direct consequence of the TCA cycle. This is unlike the mammalian TCA cycle where the isocitrate dehydrogenase enzyme, Idh1p, generates NADPH.

**Figure 4:**
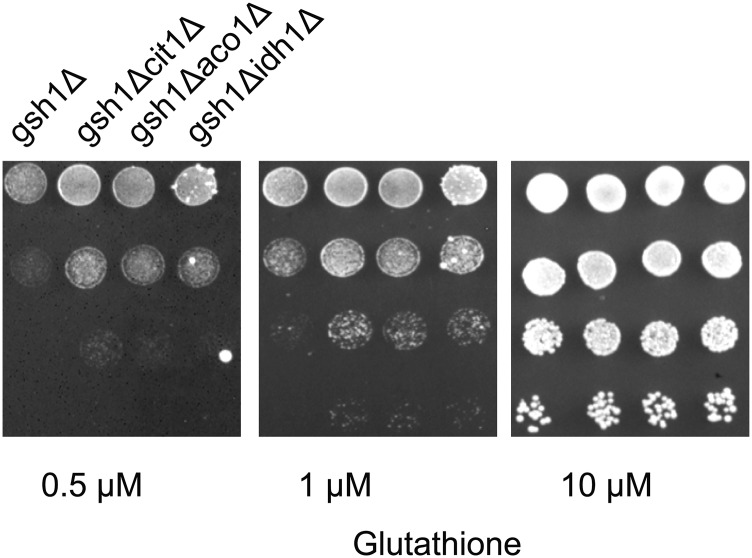
Growth of deletions of selected TCA cycle enzymes in *gsh1Δ* background at different glutathione concentrations. Growth and serial dilution of *cit1Δgsh1Δ*, *aco1Δgsh1Δ*, and *idh1Δgsh1Δ* performed as described in legends to Figure (1A).

We decided to evaluate the genetic screen under conditions where any of the initial enzymes of the TCA cycle were knocked out in a *gsh1∆* background. Citrate synthase, Cit1p is the first enzyme of the TCA cycle where the incoming Acetyl CoA is coupled to oxaloacetate to form citrate. Aconitase, Aco1p, converts citrate to isocitrate, while Isocitrate dehydrogenase, Idh1p, oxidizes isocitrate to form α-ketoglutarate while also generating NADH. When we evaluated each of these knockouts in a *gsh1∆* background we found that these mutants showed enhanced growth relative to *gsh1∆* (Figure 4). We have not investigated the biochemical basis of these phenotypes, However, as it is known that knockout of these TCA enzymes can be a signal for oxidative stress (Lushchak *et al.* 2014), it is possible that the knockouts of these enzymes are pushing the oxidative branch of the pentose phosphate pathway to generate more NADPH.

### Exploiting the genetic screen for evaluation of mitochondrial genes important for NADPH homeostasis

The evaluation and validation of the screen with enzymes specifically altering NADPH levels either directly or indirectly, prompted us to set up a more detailed analysis to identify new participants that might affect NADPH homeostasis. Since mitochondrial NADPH generation is less well understood, we decided to investigate genes that encode proteins localized to the mitochondria. We targeted both known and unknown proteins that are reported to be localized to the mitochondria. As a starting point, we began with all the genes from the *Saccharomyces* Genome Database (SGD) that were picked up using mitochondria as a keyword in the search criteria. A total of 369 genes were identified. Of these 129 genes were essential and were thus not pursued in this analysis. We focused on the 240 genes that were non-essential. We procured strains from Euroscarf with deletion in each of these 240 genes, and deleted the GSH1 gene in these mitochondrial gene deletion backgrounds. The *gsh1* deletions were confirmed by glutathione auxotrophy; these deletions were then evaluated in our screen using suboptimal glutathione concentrations in the media. The serial growth experiments were repeated at least twice for all strains. Of the total of 239 genes, we found that *gsh1∆* in 189 gene deletion backgrounds showed no significant difference in growth relative the *gsh1∆* strain, *gsh1∆* in 35 gene deletion strains showed improved growth as compared to *gsh1∆* alone, while *gsh1∆* in 15 gene deletion strains showed decreased growth as compared to *gsh1∆* alone (Supplementary Table no. 1 and Supplementary 2).

**Figure 5:**
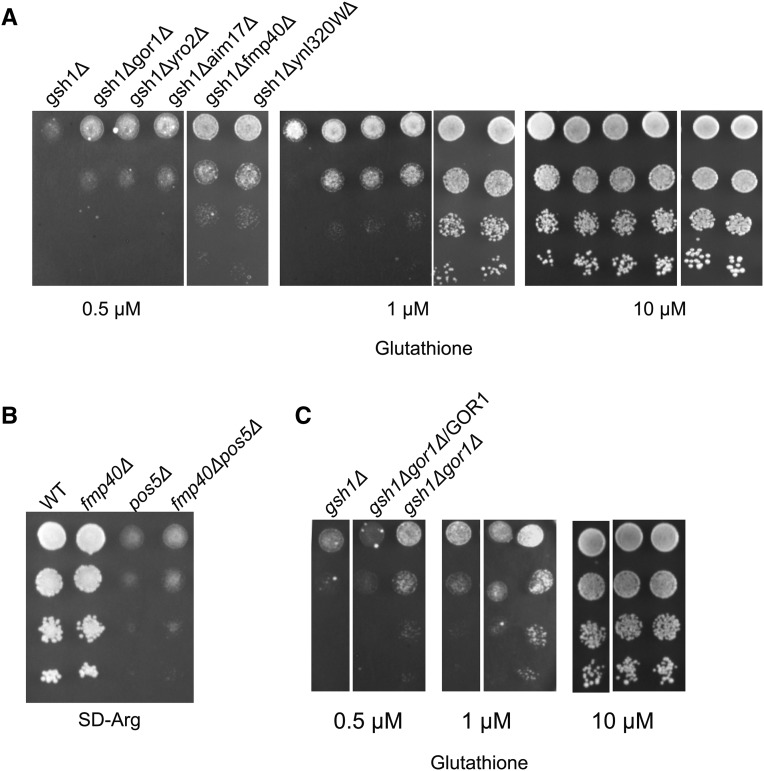
(A) Growth of deletions of selected mitochondrial gene deletions in *gsh1Δ* background showing improved growth in the screen Growth and serial dilution of *gor1ΔΔgsh1Δ*, *yro2Δgsh1Δ*, *ynl320wΔgsh1Δ*, *fmp40Δgsh1Δ*, *aim17Δgsh1Δ* performed as described in legends to Figure (1A). (B) Complementation of *gor1Δ* phenotype in *gsh1Δ* background by the WT GOR1 clone. (C) Growth of *fmp40Δ*, *pos5**Δ* and *fmp40Δ**pos5**Δ* potential suppressor of arginine auxotroph in *fmp40Δpos5Δ*.

The list included many known and unknown or poorly characterized genes. Some of them could also be resulting in ROS generation that would lead to increased flux into the pentose phosphate pathway, but this needed to be examined. Among the genes whose knockout in the *gsh1∆* background conferred significantly better growth included FMP40, YRO2, GOR1 and AIM17 (Supplementary Table no. 1 and Figure 5A).

### Fmp40p is a strong consumer of NADPH and the fmp40Δ can rescue the deletion phenotypes of the mitochondrial NAD(H) kinase, Pos5p

FMP40 was one of the mitochondrial genes whose deletion showed significantly improved growth in the glutathione limiting screen. The phenotype was confirmed by complementing the deletion with a WT copy of the FMP40 gene (data not shown). Very recently, Fmp40p, the yeast homolog of human Selenoprotein O, has been reported to function as a mitochondrially-localized pseudokinase (Sreelatha *et al.* 2018) where it AMPylates specific proteins such as glutaredoxins as a means to counter oxidative stress. Interestingly, the AMPylation activity of Fmp40p was shown to occur after the disulfide bond of Fmp40p was reduced. *In vitro* studies showed that Fmp40p reduction could occur by DTT or by NADPH. Although the function of the Fmp40p was clearly delineated, the *in vivo* reductant of Fmp40p required for its activation was not clear. Further, the requirement for a reductive step to activate a protein required to counter oxidative stress was somewhat surprising, and revealed additional roles for the reductive activation step that were not clear. We therefore decided to examine using our genetic screen, whether NADPH was the true reductant of FMP40.

If FMP40 was being reduced by NADPH then the significant increase in NADPH levels that would result from the *fmp40Δ* phenotype, would also be able to suppress other mitochondrial defects that were leading to low NADPH. Pos5p is a mitochondrial NAD(H) kinase responsible for the significant NADPH levels in the mitochondria (Pain *et al.* 2010). The initial steps of arginine biosynthesis occur in mitochondria and one of the early steps involves NADPH reduction. Owing to the deficiency of NADPH in *pos5**Δ* strains, the *pos5**Δ* strains are auxotrophic for arginine (Outten and Culotta 2003; Bieganowski *et al.* 2006). We therefore evaluated if *fmp40Δ* might at least partially suppress the *pos5**Δ* slow growth phenotype, and the arginine auxotrophy. After creating an *fmp40Δ* in a *pos5**Δ* strain, we evaluated the growth of the strains and found a significant improvement in growth and partial reversion of arginine auxotrophy of *pos5**Δ* in an *fmp40Δ* deletion background as compared to WT background (Figure 5B). This result suggested that the natural reductant for Fmp40p was indeed NADPH.

### Gor1p is a strong consumer of NADPH and has activity primarily against glyoxylate (as opposed to hydroxypyruvate) but blocking glyoxylate formation could not rescue the phenotype

**Figure 6:**
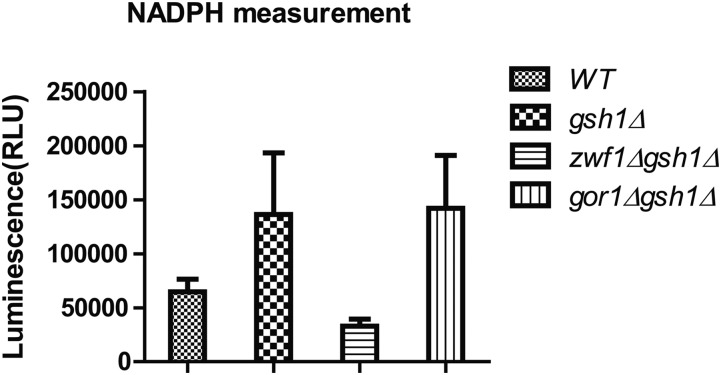
Estimation of NADPH on different strains WT, *gsh1Δ*, *zwf1Δgsh1Δ* and *gor1Δgsh1Δ* Cells were grown in minimal media and extracts assayed for NADPH by luminescence-based method using NADP/NADPH-Glo assay kit as indicated in materials and methods.

GOR1 encodes a glyoxylate reductase/hydroxypyruvate reductase and is a known NADPH consuming enzyme. Deletion of GOR1 also showed a significant improvement in the growth of *gsh1∆*, suggesting that it is a strong consumer of NADPH. We first determined that the phenotype was genuine by confirming that the WT GOR1 gene could complement the deletion phenotype (Figure 5C). To determine where Gor1p is located, we first examined the sequence of Gor1 protein for a putative mitochondrial targeting sequence. Using different prediction tools (*e.g.*, MitoFates, and MitoProt II etc.) did not reveal an apparent signal sequence (Claros 1995; Fukasawa *et al.* 2015). We therefore tagged the protein and carried out experiments to determine the primary localization. Our results indicated that the Gor1p was localized primarily in the mitochondria but a very small fraction seemed to be also present in the cytoplasm (Supplemental Material, figure S1). Interestingly, high throughput studies on non-tagged proteins have also indicated Gor1 protein as a mitochondrial localized protein. (Sickmann *et al.* 2003; Reinders *et al.* 2006). We also estimated the relative NADPH levels in the *gor1∆* strains and the control *gsh1∆* backgrounds, using a luminescence-based biochemical kit for NADPH estimation (Bankapalli *et al.* 2015). *gsh1∆* cells showed increased NADPH levels compared to the wild type, indicating that low glutathione levels were being tackled by the cells through NADPH compensation. Further, *zwf1∆* also showed the expected lower levels of NADPH. However, the *gor1∆gsh1∆* cells showed only comparable or marginally higher levels of NADPH (Figure 6). It is possible that the increase in NADPH levels that was occurring might be specifically occurring in the mitochondrial compartment, which was not being reflected in the overall NADPH levels.

Yeast Gor1p was initially reported to be a glyoxylate reductase (Rintala *et al.* 2007). However, a detailed sequence analysis of the hydroxypyruvate/glyoxylate reductase family has revealed that there are two distinct branches in the family, and based on sequence analysis, yeast GOR1 was in fact placed in the hydroxypyruvate reductase family (Kutner *et al.* 2018). We therefore decided to reinvestigate the protein and the phenotype more carefully.

**Figure 7:**
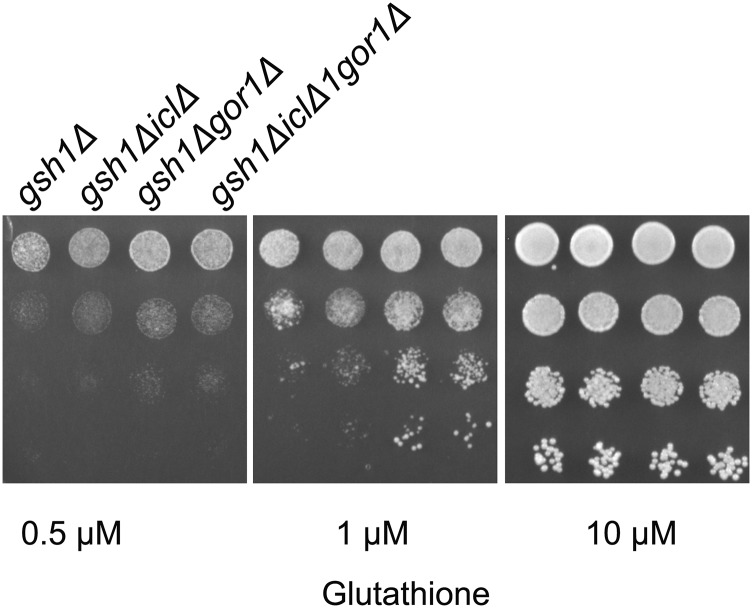
Comparison of deletions of *icl1Δ*, in *gor1Δgsh1Δ* background Growth and serial dilution of *gsh1Δ*, *icl1Δgsh1Δ* and *icl1Δgor1Δgsh1Δ* performed as described in legends to Figure (1A).

**Figure 8:**
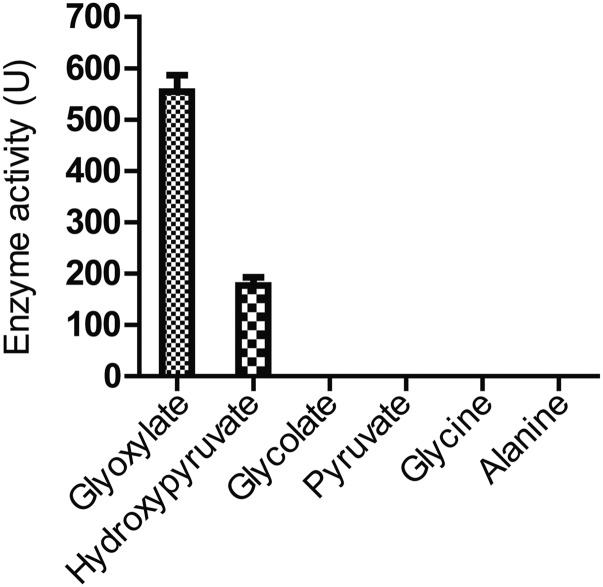
Relative activity of Gor1p toward different substrates.

The enzyme which is thought to be the key enzyme required for the formation of glyoxylate in the glyoxylate pathway is Isocitrate lyase, Icl1p which is cytosolically localized. ICL1 is also repressed under glucose grown conditions. As the experiments we had carried out were under glucose containing media, we were curious to examine if the improved growth on *gsh1∆* we were observing was Icl1p dependant. We, therefore, created an ICL1 deletion in the *gor1Δgsh1∆* background. In case glyoxylate was the substrate of Gor1p then its generation through Icl1pwould be blocked and we would not see the enhanced growth upon *gor1Δ* in the *gsh1∆* background. However, when we deleted ICL1 in the *gor1Δgsh1∆* background no suppression of the *gor1Δ* phenotype was observed (Figure 7). This suggested that either glyoxylate was not the true substrate of Gor1p, or there was as an alternate route for glyoxylate formation. Since no alternate formation of glyoxylate has been reported, we considered the possibility that glyoxylate was not the true substrate of glyoxylate; this was consistent with the placement of yeast GOR1 on a hydroxyl pyruvate reductase (Kutner *et al.* 2018). To examine this more carefully, we purified the Gor1p through expression in *E. coli* and the purified protein was examined for activity against hydroxypyruvate *vs.* glyoxylate. We observed a preferential activity toward glyoxylate as a substrate, consistent with an earlier report (Rintala *et al.* 2007). The *Km* of the Gor1p enzyme toward glyoxylate was 8.4 mM while toward hydroxypyruvate it was 22.7 mM (data not shown). Despite the preferential activity of Gor1p toward glyoxylate, the *Km* was in the millimolar concentration, and there are no reports that glyoxylate accumulates at these high levels. This is especially the case when experiments are being done in glucose grown media, conditions where ICL1 is repressed. We, therefore, checked alternate available substrates such as pyruvate, glycine, alanine and glycolate that might also be expected to accumulate at high levels. However, among the various substrates examined, we could not find significant activity of the enzyme toward these substrates (Figure 8). It is possible therefore that some other unknown metabolite might be also a substrate for Gor1p in yeast cells. Notwithstanding the lack of clarity on the true substrates of Gor1p, the enzyme was clearly a major contributor to the consumption of NADPH in cells under these conditions.

## Discussion

In this manuscript, we describe the development of a simple and robust genetic screen for factors that influence the growth of yeast cells under sub-optimal glutathione conditions. As NADPH is expected to play a major role under these conditions, the genes and mutations that we identify are likely to be those affecting NADPH metabolism in the cell.

The screen was validated by first evaluating genes and pathways that are known to both directly and indirectly affect NADPH levels. Once this was confirmed, we employed the genetic screen to screen the mitochondrial gene deletion collection. This was done by introducing a *gsh1∆* in these mitochondrial gene deletion background and then evaluating them for growth on suboptimal concentrations of glutathione. The mitochondrial gene set that we have chosen, however, probably does not represent the full set of genes encoding proteins targeted to the mitochondria, since a recent study has indicated that the yeast mitochondrial proteome includes 750 proteins, whereas what we have targeted here is only 369 genes.

Following the systematic analysis of the disruptants in the screen, we discovered several new players and following up on these in subsequent studies could be rewarding. For the present study, however, we restricted the study to two gene deletions, FMP40 and GOR1 that showed significantly improved growth in the assay. At the time we initiated the work the function of FMP40 was still unknown, while GOR1 had ambiguous functions. Both these gene products had interesting aspects, and the genetic screen allowed us to further investigate the role of these genes in NADPH homeostasis. Both also appeared to be the major consumers of NAPDH, at least under the screen conditions employed.

One of the features of the recently described functions of Fmp40p is that the protein is involved in AMPylation of proteins such as glutaredoxins to protect cells from oxidative stress (Sreelatha *et al.* 2018), and yet, surprisingly requires reduction of a disulfide bond by NADPH for its activation. A reductive step to activate proteins facing oxidative stress is somewhat surprising. Considering that this protein is also one of the major consumers of NADPH, it seems likely that the reductive step might also be a sensor for NADPH/NADP^+^ ratios.

Gor1p, which was also found to be a major consumer of NADPH in our screen, has been previously suggested to function as a sink for NADPH in plants (Nelson and Tolbert 1970; Allan *et al.* 2009). Glyoxylate, an aldehyde is toxic to DNA and needs to be removed from the cell. The removal has been shown to be dependent on the NADPH/NADP^+^ ratios (Allan *et al.* 2009). Thus, while removing the toxic metabolite it can also act as a sink of excess NADPH during stress conditions. During low glutathione conditions, NADPH levels are high, and a sink is needed for removal of excess electrons. However, the true substrate of Gor1p remains a matter of debate, both from deletion of the enzyme Icl1p (known to generate glyoxylate), and from the enzyme kinetics of Gor1p (which revealed a high *Km* for glyoxylate).

The expression of GOR1 has been reported to depend upon conditions the mimic oxidative stress conditions, under which NADPH levels can also rise significantly (Larochelle *et al.* 2006; Dephoure and Gygi 2012; Bergman *et al.* 2019; Li *et al.* 2019). One possibility, therefore, is that to tackle these excess electrons, Gor1p can act as a sink for NADPH, ensuring not only the removal of glyoxylate, but also removing the excess NADPH. Clearly more studies are required to understand the interplay of these enzymes and NADPH homeostasis.
